# Evaluating Change in Student Pharmacists’ Familiarity, Attitudes, Comfort, and Knowledge as a Result of Integrating Digital Health Topics Into a Case Conference Series: Cohort Study

**DOI:** 10.2196/43313

**Published:** 2023-07-10

**Authors:** Julia C Darnell, Mimi Lou, Lisa W Goldstone

**Affiliations:** 1 College of Pharmacy Western University of Health Sciences Pomona, CA United States; 2 Alfred E. Mann School of Pharmacy and Pharmaceutical Sciences University of Southern California Los Angeles, CA United States

**Keywords:** digital health, telehealth, digital therapeutics, mobile health applications, wearable health technologies, pharmacy education, medical education, patient cases, technology, education, digital, digital health, survey, intervention

## Abstract

**Background:**

The use of technology in health care, often referred to as digital health, has expanded rapidly because of the need to provide remote care during the COVID-19 pandemic. In light of this rapid boom, it is clear that health care professionals need to be trained in these technologies in order to provide high-level care. Despite the growing number of technologies used across health care, digital health is not a commonly taught topic in health care curricula. Several pharmacy organizations have called attention to the need to teach digital health to student pharmacists; however, there is currently no consensus on best methods to do so.

**Objective:**

The objective of this study was to determine if there was a significant change in student pharmacist scores on the Digital Health Familiarity, Attitudes, Comfort, and Knowledge Scale (DH-FACKS) after exposure to digital health topics in a yearlong discussion–based case conference series.

**Methods:**

Student pharmacists’ initial comfort, attitudes, and knowledge were gathered by a baseline DH-FACKS score at the beginning of the fall semester. Digital health concepts were integrated into a number of cases in the case conference course series throughout the academic year. The DH-FACKS was administered again to students after completion of the spring semester. Results were matched, scored, and analyzed to assess any difference in DH-FACKS scores.

**Results:**

A total of 91 of 373 students completed both the pre- and postsurvey (response rate of 24%). Using a scale from 1 to 10, the mean student-reported knowledge of digital health increased from 4.5 (SD 2.5) before intervention to 6.6 (SD 1.6) after intervention (*P*<.001) and the mean self-reported comfort increased from 4.7 (SD 2.5) before intervention to 6.7 (SD 1.8) after intervention (*P*<.001). There was a significant increase in scores for all 4 elements of the DH-FACKS. The mean familiarity scores increased from 11.6 (SD 3.7) to 15.8 (SD 2.2), out of a maximum of 20 (*P*<.001). The mean attitudes scores increased from 15.6 (SD 2.1) to 16.5 (SD 1.9), out of a maximum of 20 (*P*=.001). The mean comfort scores increased from 10.1 (SD 3.9) to 14.8 (SD 3.1), out of a maximum of 20 (*P*<.001). The mean knowledge scores increased from 9.9 (SD 3.4) to 12.8 (SD 3.9), out of a maximum of 20 (*P*<.001).

**Conclusions:**

Including digital health topics in a case conference series is an effective and approachable way of providing education on important digital health concepts to students. Students experienced an increase in familiarity, attitudes, comfort, and knowledge after the yearlong intervention. As case-based discussions are an important component of most pharmacy and other medical curricula, this method can be easily applied by other programs that wish to give their students practice applying their knowledge of digital health to complex case-based scenarios.

## Introduction

### Background

Digital health is a topic of increasing interest in the medical field, especially in light of the COVID-19 pandemic, and a push to increase remote care and use digital medicine [1,2]. In September 2020, the Food and Drug Administration launched the Digital Health Center of Excellence with the goal to “empower stakeholders to advance health care by fostering responsible and high-quality digital health innovation” [3]. Digital health is a broad term that encompasses many topics including mobile health apps, digital therapeutics, wearable health technology, artificial intelligence, health information technology, and telehealth. Considering the increasing number of wearable health technologies, mobile health apps, and digital therapeutics being produced, and in some cases approved by the Food and Drug Administration, it is important that health care workers, including trainees, be equipped with the skills to understand and apply digital health to optimize patient care. There have been several studies gauging perceptions and competencies in digital health in medical training curricula [4-7]. These studies have shown that, in general, students recognized the advantage of integrating digital health into patient care; however, the majority of students rated their digital health skills as poor.

In 2017, the International Pharmaceutical Federation (FIP) released a report addressing the need to incorporate digital health education into pharmacy curricula [8]. The FIP surveyed pharmacy schools worldwide, and the results showed that only 43% of schools included digital health in their curricula. The majority of institutions that did have digital health as part of their curriculum reported a low frequency of digital health exposure, with 35% of respondents reporting only 1 to 2 lectures given in an academic year. Of the students who responded to the FIP survey, only 10% reported learning digital health in their pharmacy curriculum [8]. Results from the FIP report indicate a clear opportunity for growth within the academic setting to prepare pharmacy learners to excel in the evolving digital health care landscape.

The American Academy of Colleges of Pharmacy (AACP) has also brought attention to the need to incorporate digital health education into pharmacy education to ensure that graduating pharmacists are educated and prepared to practice in an increasingly digital health care world. After the release of the FIP report, AACP highlighted several institutions that have spearheaded digital health education [9]. AACP also held a digital health institute in October 2021 to help pharmacy programs develop a plan to incorporate emerging health care technologies into their respective curricula. These efforts have brought together experts and pioneers in digital health and pharmacy education to share ideas and empower educators to incorporate digital health at their institutions. This call to educate student pharmacists in digital health to prepare them for careers of the future continues to be echoed in educational literature [7,10-13].

Despite the call to action for digital health pharmacy education from FIP and AACP, there is currently no consensus on best practices to do so, although several methods for integrating digital health into pharmacy education and training have been proposed [10,12]. One process is to incorporate digital health throughout the entirety of the pharmacy curriculum in didactic, laboratory, and experiential settings. Other approaches include instating a digital health elective, offering a separate digital health certificate or degree, or a capstone project in digital health. A program can also choose to use multiple methods within their curriculum. Although there is no widely accepted methodology to providing digital health education in medical education [12], one digital health expert has commented that weaving digital health throughout the continuum of the curriculum would be ideal rather than siloing it into one course or an elective track [14]. As the opportunities for pharmacists to use digital health in their practice are expanding [15-20], it is important that digital health be highlighted in a variety of settings and topics. By incorporating digital health into a variety of courses through the duration of a student’s education, this ensures that digital health education does not occur on one isolated occasion and is delivered to all students and not just a select few.

### Study Objective

Before this study, digital health had not been formally taught or assessed in the University of Southern California (USC) PharmD curriculum. To address the need to weave digital health into the pharmacy curriculum, the USC School of Pharmacy proposed several strategies to integrate digital health throughout the curriculum. The first step was incorporating digital health topics into the required case conference series, which runs concurrently with therapeutic courses for a total of 4 semesters during the second (P2) and third year (P3) of a 4-year PharmD program. The case conference course is a 2-credit unit, discussion-based course that runs parallel to didactic pharmacotherapy courses. Each week students are assigned a case and prework to review before the active learning case session. Topics covered in the 2021-2022 case conference included clinical cases focused on medication therapeutic management, drug information questions, ethical dilemmas, population health evaluation, pharmaceutical industry topics, and digital health. Because of the highly active and discussion-based nature of case conference, it was decided that this would be an optimal setting to first integrate digital health into the pharmacy curriculum in a longitudinal manner. Using the currently existing case conference series allowed for the flexibility to teach digital health without requiring additional teaching hours being added to the curriculum.

The objective of this study was to assess change in student pharmacists’ familiarity, attitudes, comfort, and knowledge (FACK) of digital health after the intentional integration of digital health topics into the case conference series. Familiarity, attitudes, and comfort were chosen as end points to assess subjective student-perceived changes related to digital health. Knowledge was assessed to determine whether there was an objective, measurable change in topic retention as a result of the educational intervention. Gathering results across these categories was determined by the study team to provide the most well-rounded and robust data to best understand the impact of the intervention.

## Methods

### Ethics Approval

The study was approved by the University of Southern California institutional review board (UP-21-00900). Students were consented into the study per the approved institutional review board protocol.

### Study Population

All P2 and P3 students enrolled in the case conference course series for the 2021-2022 academic year were eligible to participate in the study. Participation in the study was voluntary and had no impact on course grades.

### Questionnaire Design and Scoring

A questionnaire, the Digital Health Familiarity, Attitudes, Comfort, and Knowledge Scale (DH-FACKS), was developed by the study team to assess the study outcomes. All questions are original to the DH-FACKS, although surveys from related studies were researched to help with survey formulation. The DH-FACKS consists of 22 questions measured by a 5-point Likert scale, single-selection multiple-choice and sliding scale, organized into 5 distinct sections. The first section includes 2 general questions asking students to rate their overall knowledge and comfort regarding digital health on a scale of 0 (no knowledge) to 10 (expert knowledge). The attitudes section prompts students to choose their level of agreeance with 4 statements about digital health. Answer choices were assigned a score as follows: strongly agree (5 points), somewhat agree (4 points), neither agree nor disagree (3 points), somewhat disagree (2 points), or strongly disagree (1 point), with the exception of one negative question where the scoring was reversed. Scores from all 4 questions were combined to determine the section total that could range from 4 to 20 points. For the familiarity section of the questionnaire, students were given a list of 10 digital health technologies and asked to select all with which they were familiar. The total number of tools the students were familiar with was calculated by counting how many tools the students selected. The students were also asked to choose their level of familiarity with 4 specific digital health topics: wearable health technology, health and wellness apps for smart devices, digital therapeutics, and telehealth. Answer choices were scored as follows: very familiar (5 points), somewhat familiar (4 points), neither familiar nor unfamiliar (3 points), somewhat unfamiliar (2 points), or very unfamiliar (1 point). Scores from all 4 questions were combined into a section total that could range from 4 to 20. For the comfort section, students were asked to rate their comfort from very comfortable to very uncomfortable, with teaching or counseling a patient on the same 4 digital health categories in the familiarity section. Scoring for the comfort section was similar to the familiarity section. For the final section, student knowledge was assessed by asking 6 multiple-choice questions created by the study team that reflected the digital health content included within the selected cases and prework. One multiple-choice question was discarded and not included into the final score, as the study team determined that the content matter of the question was not best suited to teach or assess in the case conference series. Students were instructed to choose the best answer from 4 answer choices in regard to the following topics: general digital health, wearable health technology, telehealth, smart medications, and the difference between mobile health apps and digital therapeutics. If the students chose the best, most complete answer choice, they received a score of 4 points; if they chose a partially correct answer, they received 2 points; and if they chose an incorrect answer, they received zero points. The section score could range from 2 to 20. Each answer was coded and scored, and a total score was calculated for each section: FACK. The DH-FACKS was housed in Qualtrics and was distributed to students via an email link unique to each participant. Presurvey data were gathered from the student baseline survey conducted at the beginning of the fall 2021 semester, and postsurvey data were gathered at the end of the spring 2022 semester.

### Intervention

For the study intervention, a total of 5 cases in the P2 and 4 cases in the P3 case conference series (due to a truncated spring semester) were chosen to include an embedded digital health topic (Textbox 1). Each of these cases included 1 learning objective and at least 1 prework assignment related to the digital health topic. At the start of the case session, students were given a 4-question quiz. In cases that incorporated digital health, one of the quiz questions was related to the digital health topic being covered. During the case session, students were prompted to discuss the digital health topic as was relevant to the case. Topics discussed included wearable health technology, mobile health apps, sensor-enabled medication devices, telehealth, and electronic health records. In the fall, both the P2 and P3 students participated in a population health case that focused on the use of digital health to develop a clinical service aimed to improve population health outcomes. For this case, a video lecture was recorded by the study author that discussed definitions of key digital health concepts, as well as examples of specific digital health tools relevant to pharmacy practice. Students were instructed to watch the video before the case to allow for optimal discussion and application to a creation of a population health clinical service.

**Timeline of the study intervention.**

**Fall semester**

- Presurvey
- Four cases incorporating digital health
- Digital health focused population health

**Spring semester**

- Five cases incorporating digital health
- Postsurvey

### Statistical Analysis

The scores from paired pre- and postsurveys were compared to determine any statistical changes in learner FACK of digital health after the integration of specific digital health topics into the yearlong course using the Wilcoxon signed rank sum test. For the Likert scale questions, responses were consolidated into 2 categories. For attitudes, strongly and somewhat agree were combined as the “positive” group, and neither agree nor disagree, somewhat disagree, and strongly disagree were combined as the “negative or neutral” group. Answer choices were combined in the same manner for familiarity and comfort questions. The categorized choices in pre- and postdata were compared using the McNemar test to determine the agreeance. A *P* value of less than .05 was considered statistically significant. All analyses were conducted using SAS (version 9.4; SAS Institute).

## Results

### Overall Change in DH-FACKS

The DH-FACKS was distributed to 373 students. A total of 91 students completed both the pre- and postsurvey (completion rate of 24%). When asked to rank their overall knowledge of digital health on a scale of 0 (no knowledge) to 10 (expert knowledge), the mean of the student-reported response significantly increased from 4.5 (SD 2.5) before intervention to 6.6 (SD 1.6) after intervention (*P*<.001). The mean of the student-reported response regarding overall comfort with using digital health in practice, using the same 0 to 10 scale, significantly increased from 4.7 (SD 2.5) before intervention to 6.7 (SD 1.8) after intervention (*P*<.001). The mean score for each section of the DH-FACKS increased after the intervention (all *P*≤.001, Table 1).

**Table 1:** DH-FACKS^a^ category scores before or after intervention.

	Prescore^b^	Postscore^b^	Difference^b^	*P* value^c^
Familiarity	11.6 (3.7)	15.8 (2.2)	4.2 (4.0)	<.001
Attitudes	15.6 (2.1)	16.5 (1.9)	0.9 (2.5)	.001
Comfort	10.1 (3.9)	14.8 (3.1)	4.7 (3.9)	<.001
Knowledge	9.9 (3.4)	12.8 (3.9)	2.9 (4.7)	<.001

^a^DH-FACKS: Digital Health Familiarity, Attitudes, Comfort, and Knowledge Scale.

^b^Data are presented as mean (SD); data do not add up to 91 because of missing data.

^c^*P* values are calculated from the Wilcoxon signed rank sum test; statistically significant at *P*<.05.

### Familiarity and Comfort

**Figure 1:**
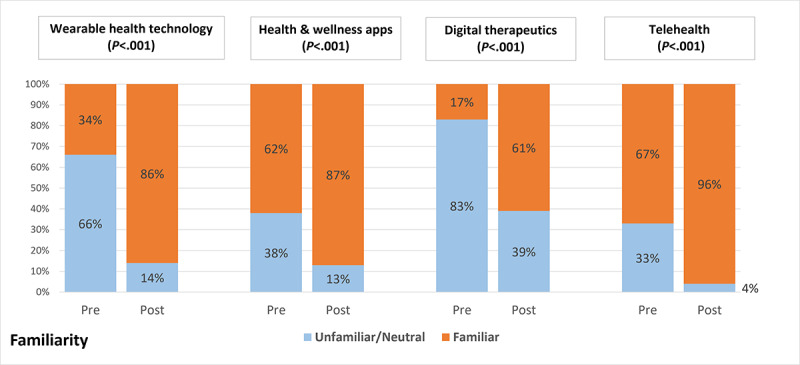
Change in familiarity with specific digital health tools.

**Figure 2:**
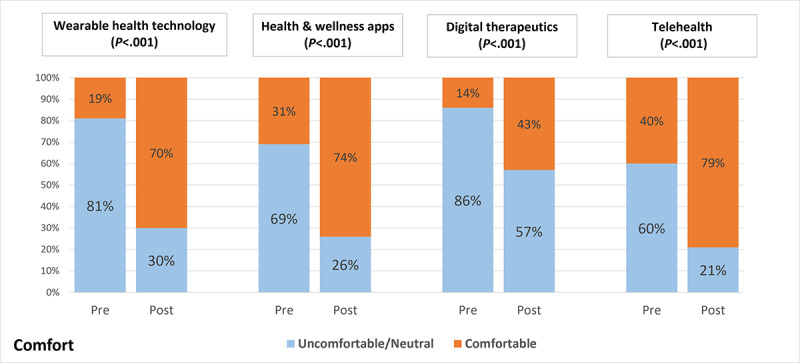
Change in comfort with specific digital health tools.

When asked to select all digital health tools they were familiar with out of a list of 10, the mean number of students selected increased from 3 (SD 1.9) to 5 (SD 1.8) after the intervention (*P*<.001). Five tools demonstrated a significant increased rate of being selected by students after the intervention: smart pills, digital therapeutics, health and wellness apps for smart devices, and telehealth. When asked to rate their level of comfort and familiarity with 4 specific tools (wearable health technology, mobile health and wellness apps, digital therapeutics, and telehealth), there were a significantly higher percentage of students who responded that they were somewhat or very familiar and comfortable with all 4 topics comparing before and after the intervention (Figures 1 and 2).

### Attitudes and Knowledge

**Figure 3:**
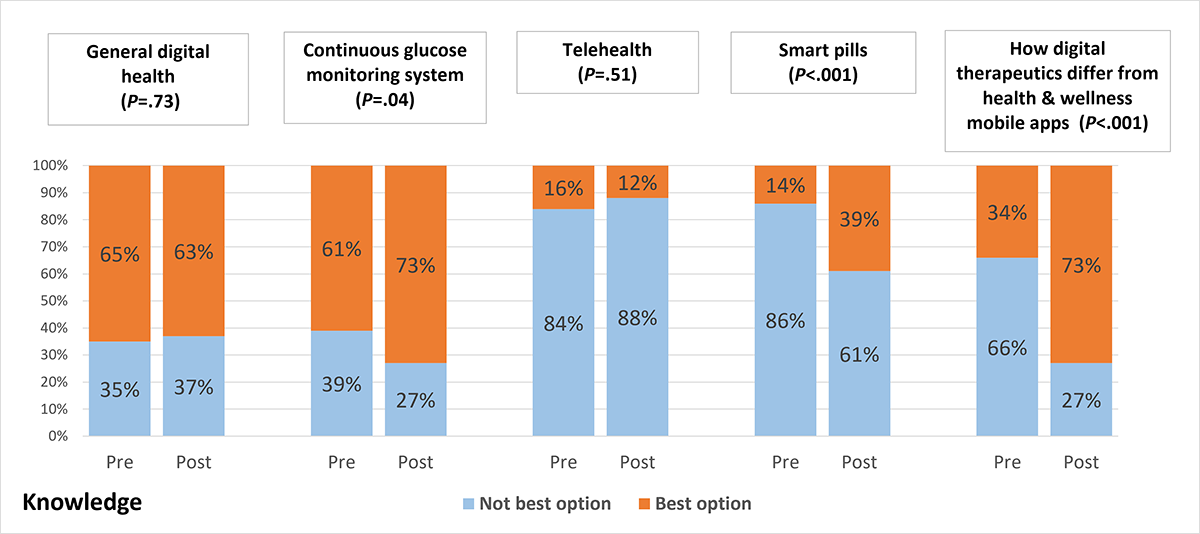
Change in digital health knowledge.

One of the 4 questions related to attitudes toward digital health observed a significant change in response after intervention. When asked to rate their agreeance with the statement “The USC curriculum has prepared me to understand concepts of digital health,” the percentage of students who either strongly or somewhat agreed with the statement significantly increased after the intervention (Table 2). The other 3 attitudes’ statements did not show a significant change in response rate after the intervention; however, high positive responses were observed in both pre- and postsurvey. A total of 3 of the 5 knowledge-based questions reported an increase in percentage of students who chose the best answer choice (Figure 3).

**Table 2:** Change in attitudes toward digital health.^a^

Pre		Post		
		Positive, n (%)	Negative or neutral, n (%)	*P* value^a^
**Q: Digital health is an important aspect of patient care**				
	Positive	81 (89)	0 (0)	N/A^b^
	Negative or neutral	10 (11)	0 (0)	N/A
**Q: I do not think digital health should be a required element of pharmacy curriculums**				
	Positive	40 (44)	15 (16)	.49
	Negative or neutral	19 (21)	17 (19)	N/A
**Q: The current USC^c^ curriculum has prepared me to understand concepts of digital health**				
	Positive	27 (30)	6 (7)	<.001^d^
	Negative or neutral	45 (49)	13 (14)	N/A
**Q: I would like to learn more about digital health**				
	Positive	40 (44)	15 (16)	.49
	Negative or neutral	19 (21)	17 (19)	N/A

^a^*P* values are calculated from the McNemar test.

^b^N/A: not applicable.

^c^USC: University of Southern California.

^d^Statistically significant at *P*<.05.

## Discussion

### Principal Findings

Results from this study show that the addition of digital health content into a case conference series led to a significant increase in all 4 categories of the DH-FACKS, with the largest increases being in familiarity and comfort. Most notably, there was an increase of familiarity and comfort with all 4 of the specific digital health categories: wearable technology, health and wellness mobile apps, digital therapeutics, and telehealth. Although there are many additional pertinent digital health topics students should be exposed to, these 4 were chosen as topics that have broad applicability to a variety of scenarios that could be integrated into patient cases.

Interestingly, students reported a significant increase in comfort and familiarity with digital therapeutics despite this not being a topic that was directly included into any of the cases. Digital therapeutics was briefly discussed in a video assigned as prework for one of the cases but was not built into any of the patient cases. This finding could suggest that even brief exposure to this topic allowed some students to grasp the basics of the topic. Conversely, this increase in familiarity and comfort could be due to some students being exposed to digital therapeutics outside of the case conference series. Although there was an increase in the number of students who reported being familiar with digital therapeutics after the intervention, the majority of students were still neutral or unfamiliar in the postsurvey. This finding will support a more targeted effort to highlight digital therapeutics in cases going forward. The other 3 topics were covered at least once in the case conference series, which supports continuing to integrate these topics into the case conference series.

The change in student attitudes, while significant, was smaller than the other 4 areas of the DH-FACKS. Baseline scores for the attitudes section were substantially higher than the other 3 sections; therefore, there was not as much room for score improvement because students already noted positive attitudes toward digital health, even before the intervention. The higher baseline attitudes score was driven by a large percentage of students agreeing that digital health is an important aspect of health care, as well as agreeing that they would like to learn more about digital health. With the majority of incoming student pharmacists belonging to “Gen Z,” the high attitudes scores may be a reflection of this generation having positive views on technology. Members of Gen Z are considered “digital natives” as they grew up using technology in their daily lives [21], so it is very plausible that their positive views on technology would translate to their professional lives.

One meaningful change in student attitudes that was captured during the study was the percentage of students who agreed that the USC curriculum prepared them to understand that concepts in digital health increased substantially after the intervention. This change suggests that the integration of digital health topics into cases was an effective method to start incorporating digital health into the curriculum and supports the continued use of this strategy going forward. However, a portion of students still disagreed with this statement, showing that there is a continued need to improve teaching digital health efforts within the curriculum to further increase the proportion of students who feel like they are being adequately prepared to understand and use digital health upon graduation.

Although the other attitudes questions did not see a significant change in response rate after the intervention, one interesting trend in the data was regarding student responses to their attitude toward digital health in patient care. All students agreed that digital health was an important aspect of patient care after the intervention. This result again helps to reinforce the need for continued integration of digital health into the curriculum, as clearly students see this topic as something that will pertain to their future careers as pharmacists in providing patient care.

Although the overall knowledge score did improve after the intervention, it should be noted that the mean knowledge score remained relatively low even after the intervention and that not all the knowledge-based questions saw an improvement in performance. The low percentage of students answering certain knowledge-based questions correctly could be due to the fact that the questions in the DH-FACKS tend to test more general knowledge than the targeted questions they received about specific tools during the case conference course series. In particular, there were a low percentage of students who chose the best definition for telehealth, even after the intervention. As telehealth is a very broad term, it is possible that students did not understand all of the various elements included within the umbrella of telehealth. Although telehealth was discussed in several cases, students did not receive any introductory lectures on digital health; therefore, they might have only focused on what was covered in case and not been aware of the various different elements of telehealth. On the basis of the students having limited baseline knowledge of digital health, they might not have been able to properly differentiate between digital health terminology enough to properly answer the questions. These potential confounding factors would support having a more structured introductory module to digital health to ensure that students have a solid baseline understanding of the subject.

A future direction for providing digital health education would include introducing the topic early in the curriculum and providing opportunities within the first year to learn more about the definitions of digital health terminology, as well as differentiation between topics. This could lead to students having a stronger baseline knowledge of digital health going in to case conference, so they can then focus on the application and discussion of the topics in detail. As digital health has applications to a broad spectrum of disease states and health care topics, future educational ventures could also include how to best teach digital health in a longitudinal manner throughout the curriculum in addition to the case conference series.

One of the limitations of this study was that a sizeable percentage of students did not complete both the pre- and postsurveys. This could be related to the voluntary nature of the study and potentially due to survey fatigue at the end of the semester when students have to fill out multiple course evaluations in a similar time period. In order to get the most meaningful results, the study team decided to only include participants who responded to both surveys to allow for pairing of the data, which substantially reduced the sample size. Although an increased sample size would have been preferred, matching the data allowed for a more powerful analysis than would have resulted from the use of a larger unpaired data set.

Another limitation of this study is that knowledge beyond the 4 questions in the DH-FACKS questionnaire was not assessed. Although there were quiz questions students answered for the cases related to digital health, the original research protocol and student consent did not include permission to access identified grade data to match with their DH-FACKS scores as the decision to include this additional layer of assessment was made after the start of the study. Future studies could include consent to obtain these data or other knowledge-based assessments including graded projects, objective structured clinical examination, or presentations. Another limitation of the study was that although digital health concepts were discussed with students in their small groups, there was no hands-on practice with the actual digital health products. This was due to financial and time constraints with obtaining digital health tools before the beginning of the academic year. Incorporating hands-on learning with digital health is an area of future study that would allow for an additional layer of experience and learning. Using a combination of prereadings and videos, small group discussions, and hands-on practice with digital health would cater to a wider variety of student learning styles.

Despite the limitations of the study, the results of the study shed valuable light on a subject that, to date, has not been widely reported in the literature. Future directions include surveying the students who remain in the didactic portion of the curriculum as they continue with the remainder of the case conference series. We anticipate that these additional data will continue to illuminate best practices in teaching digital health to student pharmacists.

### Conclusions

Inclusion of digital health topics into a case conference course series served as an effective way of increasing student FACK with digital health and may be a valuable method for other PharmD programs to use. Although integration into cases served as a good starting point, it should be noted that inclusion of digital health in cases alone might not be sufficient to fully expose students to the breadth of important topics as shown by low knowledge scores even after the intervention. Further incorporation into the curriculum at large, including within therapeutics courses, may best serve students to understand the broad applicability of digital health to pharmacy practice.

## Abbreviations

AACP: American Academy of Colleges of Pharmacy
DH-FACKS: Digital Health Familiarity, Attitudes, Comfort, and Knowledge Scale
FACK: familiarity, attitudes, comfort, and knowledge
FIP: International Pharmacy Federation
USC: University of Southern California
